# CD10 and osteopontin expression in dentigerous cyst and ameloblastoma

**DOI:** 10.1186/1746-1596-6-44

**Published:** 2011-05-24

**Authors:** Shaimaa M Masloub, Adel M Abdel-Azim, Ehab S Abd Elhamid

**Affiliations:** 1 Oral Pathology Department, Faculty of Dentistry, Ain Shams University, Cairo, Egypt

## Abstract

**Aims and Objectives:**

To investigate the expression of CD10 and osteopontin in dentigerous cyst and ameloblastoma and to correlate their expression with neoplastic potentiality of dentigerous cyst and local invasion and risk of local recurrence in ameloblastoma.

**Methods:**

CD10 and osteopontin expression was studied by means of immunohistochemistry in 9 cases of dentigerous cysts (DC) and 17 cases of ameloblastoma. There were 7 unicystic ameloblastoma (UCA) and 10 multicystic ameloblastoma (MCA). Positive cases were included in the statistical analysis, carried on the tabulated data using the Open Office Spreadsheet 3.2.1 under Linux operating system. Analysis of variance and correlation studies were performed using "R" under Linux operating system (R Development Core Team (2010). Tukey post-hoc test was also performed as a pair-wise test. The significant level was set at 0.05.

**Results:**

High CD10 and osteopontin expression was observed in UCA and MCA, and low CD10 and osteopontin expression was observed in DC. Significant correlation was seen between CD10 and osteopontin expression and neoplastic potentiality of DC and local invasion and risk of recurrences in ameloblastoma.

**Conclusions:**

In DC, high CD10 and osteopontin expression may indicate the neoplastic potentiality of certain areas. In UCA & MCA, high CD10 and osteopontin expression may identify areas with locally invasive behavior and high risk of recurrence.

## Background

Dentigerous cyst (DC) is the most common developmental cyst in the oral cavity, accounting for 20% of the developmental cysts of the jaws, and is almost always associated with the crown of a tooth attached to the cemento-enamel junction. It is believed to originate from the accumulation of fluid between the reduced enamel epithelium and the tooth crown, thus expanding the follicle beyond the 3 mm normal diameter and hence is usually associated with impacted or un-erupted teeth [1].

Ameloblastoma is an uncommon benign, locally aggressive odontogenic neoplasm that accounts for approximately 10% of all tumors that arise in the mandible and maxilla [2]. Although the etiology is unknown, ameloblastoma is believed to develop from various sources of odontogenic epithelia, including dental follicular lining epithelium [3]. Ameloblastoma is classified as central or peripheral. Central ameloblastomas are classified as multicystic/solid (MCA) or unicystic (UCA) [4]. Multicystic/solid ameloblastomas tend to be more aggressive and have a higher likelihood of recurrence after surgery compared with unicystic and peripheral ameloblastomas [5].

CD 10 cell surface glycoprotein was initially identified as a 100 KDa tumor associated antigen (common acute lymphoblastic leukemia antigen, CALLA) on human acute lymphoblastic leukemias and other lymphoid malignancies with an immature phenotype [6]. CD10 is expressed on the surface of a variety of normal and neoplastic hematopoietic and lymphoid cells, including lymphoid precursor cells, germinal center B lymphocytes and some epithelial cells [7].

The specialized effects of the action of CD10/Neutral Endopeptidase (NEP) are attributable to the substrates present in different tissues, at points of contact between cells and extracellular matrix or between adjacent cells; surface CD10 would then regulate the local concentrations of specific peptides [8]. CD10 may also play an important role in maintenance of homeostasis, neoplastic transformation, and tumor progression [9]. Recent works suggested that CD10 expression in cancer cells could have a role both in apoptosis and proliferation [10], while CD10 expression in intratumoral stromal cells may also contribute to tumor progression [11]. High CD10 expression was associated with poor prognosis in various tumors like breast carcinoma [11], malignant melanoma [12], cutaneous basal cell and squamous cell carcinoma [13] and oral squamous cell carcinoma [14].

Meanwhile, osteopontin (OPN) was identified independently, together with bone sialoprotein (BSP), as a major sialoprotein in the extracellular matrix of bone and the 2 proteins were initially called bone sialoprotein (BSP I) and bone sialoprotein II (BSP II), respectively [15]. Constitutive expression of OPN exists in several cell types but induced expression has been detected in T lymphocytes, epidermal cells, bone cells, macrophages, and tumor cells in remodeling processes such as inflammation, ischemia-reperfusion, bone resorption, and tumor progression [16].

OPN has been shown to be multifunctional, with activities in cell migration, cell survival, inhibition of calcification, regulation of immune cell function, and control of tumor cell phenotype [17]. Binding of OPN to tumor cell membrane receptor CD44v6 can enhance tumor cell motility [18] and increase the immune adaptation of OPN-expressing cells [19]. OPN can also trigger integrin-mediated signal transduction, which, in turn, leads to osteoclast activation [20]. Ligation of OPN with integrin α5β3 on vascular endothelial cells induces neovascularization by up-regulating endothelial cell migration, survival, and lumen formation during angiogenesis [21].

High OPN expression was associated with poor prognosis in various tumors like prostate carcinoma [22], breast carcinoma [23] and cutaneous squamous cell carcinoma [24]. Concerning ameloblastoma, high OPN expression was reported in both unicystic and multicystic ameloblastoma [25].

The present study was conducted to investigate the expression of CD10 and osteopontin in dentigerous cyst and ameloblastoma and to correlate their expression with neoplastic potentiality of dentigerous cyst and local invasion and risk of local recurrence in ameloblastoma.

## Methods

### 1. Materials

Twenty-six formalin fixed paraffin-embedded archival blocks of dentigerous cyst and ameloblastoma were obtained from the archives of the oral pathology department, Ain Shams University and National Cancer Institute, Cairo University. Data of the archival paraffin blocks included the histopathological diagnosis of each case as well as history of recurrence in cases diagnosed as multicystic ameloblastoma. Each case was then coded and patient's name was not shown for ethical reasons. Nine cases were diagnosed as dentigerous cyst, seven cases were diagnosed as unicystic ameloblastoma, and ten cases were diagnosed as multicystic ameloblastoma. To confirm the diagnosis 5 µm thick sections were cut and mounted on glass slides, sections were stained with haematoxylin and eosin stain and examined by light microscope.

### 2. Immunohistochemical procedures

For all specimens 4 µm sections were cut and mounted on positively charged glass slides. Sections were deparaffinized with xylene and rehydrated in graded ethyl alcohol, and sections were immersed in citrate buffer solution of pH 4.8 and were put in the microwave oven before staining procedures. For immunostaining a universal kit (Lab Vision) was used, peroxidase anti- peroxidase method of immunostaining using the streptavidin-biotin system was carried out, and 3% hydrogen peroxide was applied to the sections to block the endogenous peroxidase activity. Sections were immunostained using the concentrated primary monoclonal antibody (clone 56C6) against CD10 (Thermo Fisher Scientific Laboratories, Ltd, United Kingdom), and monoclonal lyophilized antibody (clone OP3N) against OPN (Visionbiosystems Novocastra™ Laboratories, Ltd, United Kingdom) and then incubated overnight at room temperature after rinsing with PBS (phosphate buffered saline) solution. Sections were then covered by the link antibody followed by the streptavidin labeling antibody; after rinsing with PBS, DAB chromogen was applied to the sections followed by counter stain, then sections were dehydrated in graded alcohol, cleared in xylene and mounted.

### 3. Assessment

For each positive section, four microscopic fields showing highest immunopositivity were selected and photomicrographs were captured at a magnification of 20X. This was performed using a video camera (C5060, Olympus, Japan) which was mounted on a light microscope (BX60, Olympus, Japan). Images were then transferred to the computer system for analysis. All the steps for immunohistochemical evaluation were carried out using image analysis software (Image J, 1.41a, NIH, USA).

### 4. Statistical Analysis

Positive cases were included in the statistical analysis, carried on the tabulated data using Open Office Spreadsheet 3.2.1 under Linux operating system. Analysis of variance and correlation studies were performed using "R" under Linux operating system (R Development Core Team (2010). Analysis Of Variance (ANOVA) test was used to test the significance of mean differences within dentigerous cyst and ameloblastoma. Post Hoc test was used when ANOVA test revealed a significant difference, to assess the significance of differences within dentigerous cyst and ameloblastoma.

The results were considered significant when P value ≤ 0.05. Welch Two Sample t-test was done to compare the expression of CD10 or OPN in primary versus recurrent ameloblastoma. The statistical tests performed included Pearson's correlation analysis to test correlation between CD10 and OPN expression in dentigerous cyst and ameloblastoma.

## Results

### A) Immunohistochemical Results

**Figure 1:**
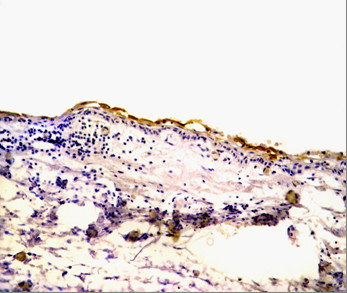
**Photomicrograph of DC showing cytoplasmic & membranous immunopositivity of superficial epithelial cells (Anti-CD10.Original magnification x40)**.

**Figure 2:**
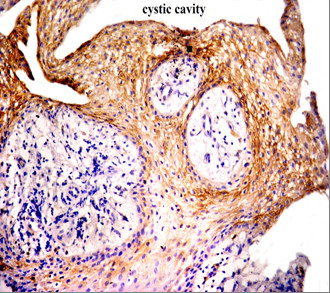
**Photomicrograph of intraluminal UCA showing cytoplasmic & membranous reaction of central cells of epithelial strands (red arrow) (Anti-CD10. Original magnification x40)**.

**Figure 3:**
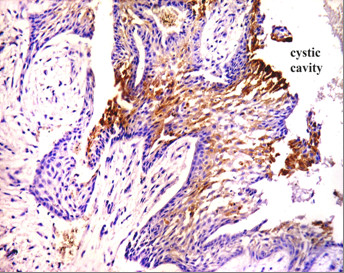
**Photomicrograph of mural UCA showing cytoplasmic & membranous immunopositivity of stellate reticulum like cells while basal & suprabasal cells are immunonegative (Anti-CD10.Original magnification x40)**.

**Figure 4:**
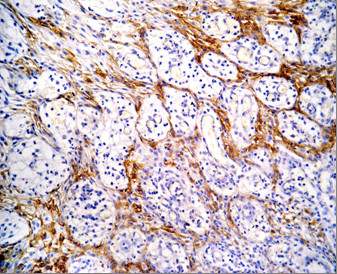
**Photomicrograph of plexiform MCA showing cytoplasmic immunopositivty of neoplastic epithelial strands (Anti-CD10.Original magnification x40)**.

**Figure 5:**
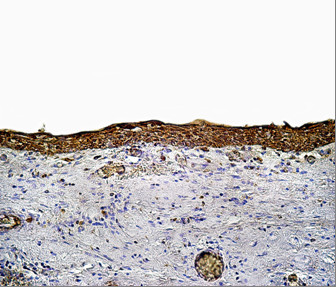
**Photomicrograph of DC showing cytoplasmic immunoreactivity in epithelial cells, inflammatory cells and in endothelial cells of blood vessels (Anti-OPN. Original magnification x40)**.

**Figure 6:**
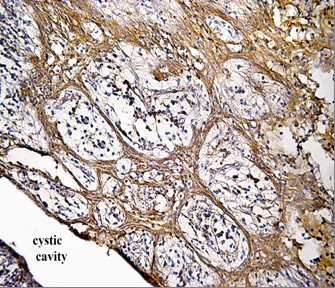
**Photomicrograph of intraluminal plexiform variant of UCA showing cytoplasmic immunopositivity in tumor cells forming strands (Anti-OPN. Original magnification x40)**.

**Figure 7:**
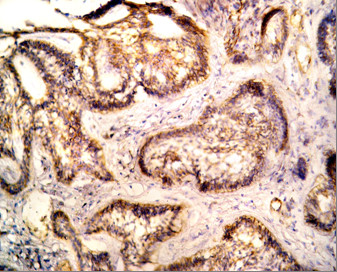
**Photomicrograph of mural follicular variant of UCA showing cytoplasmic immunopositivity in ameloblast like cells & few stellate reticulum like cells. Note the peritumoral positive reaction (Anti-OPN. Original magnification x40)**.

**Figure 8:**
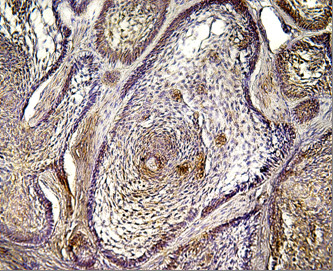
**Photomicrograph of follicular variant of MCA showing cytoplasmic immunopositivity of both ameloblast like cells & stellate reticulum like cells. Note the immunonegativity of most of the ameloblast like cells lining the large-sized follicles and peritumoral reaction (AntiOPN. Original magnification x40)**.

Positive CD10 and OPN staining was found in all cases examined in the present study. The positive cases showed a homogenous and brownish immunostaining. For CD10: In DC, cytoplasmic & membranous immunoreactivity was seen mainly in the superficial layers of the epithelial lining (Figure 1). In the intraluminal UCA, most of the central cells of epithelial strands showed membranous reaction, while few of these cells showed both membranous and cytoplasmic reaction (Figure 2). On the other hand, in the mural cases, the epithelial cells facing the cystic cavity showed both membranous and cytoplasmic reaction, while basal and suprabasal cells were immunonegative. The stellate reticulum like cells in the mural strands showed cytoplasmic reaction (Figure 3). In MCA, the neoplastic epithelial cells showed cytoplasmic and membranous immunopositivity (Figure 4). For OPN: In DC, cytoplasmic immunoreactivity was seen in epithelial cells, inflammatory cells and endothelial cells of blood vessels (Figure 5). In intraluminal UCA, the tumor cells and inflammatory cells in the stroma showed cytoplasmic reaction (Figure 6). In mural UCA, cytoplasmic immunopositivity in ameloblast like cells & few stellate reticulum like cells was seen resulting in a peritumoral positive reaction (Figure 7). In MCA, few stellate reticulum like cells showed cytoplasmic reaction, while most of the ameloblast like cells showed cytoplasmic reaction. Peritumoral reaction was also seen in the stroma surrounding the tumor (Figure 8).

### B) Statistical Results

Tukey pair wise test revealed a statistically significant difference in the expression of CD10 in DC & MCA, and in UCA & MCA (p values less than 0.001). On the other hand, there was no statistically significant difference in the expression of CD10 in UCA & DC (Table 1).

**Table 1:** Descriptive statistics (CD10)

Lesions	Mean		Std.Deviation	Std.Error
DC	3.033		2.343	36

MCA	9.995		4.915	40

UCA	4.925		2.851	28

*Tukey pair-wise test for CD10.*				

**Lesion**	**diff**	**Lower**	**upper**	**p-value**

MCA-DC	6.962	4.838	9.085	0.000

UCA-DC	1.892	-0.438	4.21	0.154

UCA-MCA	-5.070	-7.348	-2.792	0.000

* The mean difference is significant at the 0.05 level

**Figure 9:**
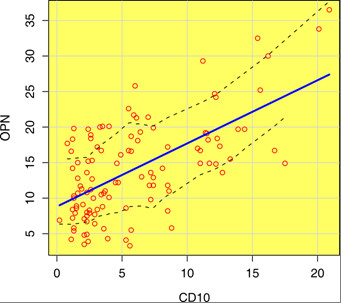
**Pearson's correlation of CD10 with OPN showing the trend line and least square lines (dotted)**.

The Tukey pair-wise test showed a statistically significant difference in the expression of OPN in MCA & DC, and in UCA & DC (p values less than 0.001). On the other hand, there was no statistically significant difference in the expression of OPN in UCA & MCA (p values more than 0.001) (Table 2). Statistical results of Welch Two Sample t-test revealed a statistically insignificant difference between CD10 or OPN mean area fraction in recurrent versus primary MCA (Tables 3 and 4). Pearson's correlation test showed a significant strong direct positive correlation between CD10 and OPN immunopositivity in either dentigerous cyst or ameloblastoma (r value = 0.622, P-value < 0,001) (Figure 9).

**Table 2:** Descriptive statistics (OPN)

lesions	Mean		Std.Deviation	Std.Error
DC	8.9		3.3	36

MCA	18.2		7.1	40

UCA	17.0		3.8	28

*Tukey pair-wise test for OPN*				

**lesion**	**diff**	**lower**	**upper**	**P-value**

MCA-DC	9.31	6.29	12.33	0.000

UCA-DC	8.14	4.83	11.45	0.000

UCA-MCA	-1.17	-4.41	2.07	0.781

* The mean difference is significant at the 0.05 level

**Table 3:** Welch Two Sample t-test (CD10)

Dependent Variable	MCA	Mean AF	95% Confidence Interval		df	P value
					
			Lower Bound	Upper Bound		
CD10 Mean Area Fraction	Primary MCA	4.6536	2.1695	3.3140	39	0.8178
					
	Recurrent MCA	4.2000				

* The mean difference is significant at the 0.05 level

**Table 4:** Welch Two Sample t-test (OPN)

Dependent Variable	MCA	Mean AF	95% Confidence Interval		df	P value
					
			Lower Bound	Upper Bound		
OPN Mean Area Fraction	Primary MCA	12.4393	3.6277	5.5415	39	0.2741
					
	Recurrent MCA	16.0667				

* The mean difference is significant at the 0.05 level

## Discussion

Dentigerous cyst (DC) is the most common developmental cyst in the oral cavity, accounting for 20% of the developmental cysts of the jaws, and is almost always associated with the crown of a tooth attached to the cemento-enamel junction [1]. Ameloblastoma is an uncommon benign, locally aggressive odontogenic neoplasm that accounts for approximately 10% of all tumors that arise in the mandible and maxilla [2]. Ameloblastoma is classified as central or peripheral. Central ameloblastomas are classified as multicystic/solid (MCA) or unicystic (UCA) [4].

The immunohistochemical results of the present study showed that cytoplasmic & membranous CD10 immunoreactivity was seen mainly in the superficial layers of the epithelial lining of DC. These results were found to be in accordance to that reported by Liapatas et al [26] who reported CD10 expression in periapical granulomas and cysts. In UCA, the results of the present study revealed that the immunohistochemical expression of CD10 in the mural cases was different from that noted in the intraluminal cases. In the intraluminal cases, CD10 was expressed in the stellate reticulum like cells and this expression was mainly membranous. On the other hand in mural cases, the epithelial cells facing the cystic cavity showed membranous and cytoplasmic immunoreactivity of CD10, while the stellate reticulum like cells in the mural strands showed mainly a cytoplasmic reaction. These results were found to be consistent with the results of other studies such as that of Granados et al. [27] and Aiad and Hanout [13] where the tumor cells in these studies showed mainly cytoplasmic expression of CD10. This difference in the expression of CD10 in the intraluminal and mural variants of UCA might explain the variable behavior of these two variants. This finding was in agreement with the argument of Rosenstein et al [28] who suggested that the luminal and intraluminal variants of UCA were non aggressive and could be treated by enucleation whereas the mural variant should be treated more aggressively and concluded that since most of the recurrent cases of ameloblastoma were of the mural type, connective tissue invasion can be considered an important microscopic sign. Linear with these data was that reported by Gardner [29] who stated that once mural invasion occurred, the UCA at this stage would act as classic intraosseous ameloblastoma and has to be treated as such. In MCA, the neoplastic epithelial cells showed cytoplasmic and membranous immunopositivity. This might be explained by the argument of Ogawa et al [30] who stated that CD10 was associated with the differentiation and growth of neoplastic cells.

Statistical analysis of the present study revealed that the CD10 mean area fraction of immunopositivity increased from DC to UCA and MCA. This could be explained by the different biological properties of CD10 which could facilitate the neoplastic transformation of DC and the locally invasive behavior of ameloblastoma. The highly aggressive and locally invasive behavior of MCA could explain the presence of a statistically significant difference in CD10 expression when comparing MCA with DC and UCA as the CD10 could facilitate this aggressive behavior.

These results were consistent with the results of Iezzi et al [31] who showed that the mean area fraction of stromal CD10 immunopositivity of MCA was higher than that of UCA and peripheral ameloblastoma. Although the mean area fraction of CD10 in UCA was higher than that of DC, this difference was found to be statistically insignificant.

The immunohistochemical results of the present study showed that OPN immunoreactivity was seen in both epithelium of DC together with underlying connective tissue. However, these results were not consistent with that reported by Wang and Liu [20] who found OPN immunopostivity in odontogenic keratocysts but not in DC. This OPN expression pattern in the epithelial lining of DC might be an early indicator of neoplastic transformation of DC into UCA. This is supported by the fact the induced expression of OPN has been detected in epidermal cells in remodeling processes as the tumor progresses [16]. Chang et al [24] further supported this argument and stated that OPN expression in both benign and malignant tumors suggested its association with the process of tumorgenesis.

The immunohistochemical results of the present study also revealed a different distribution pattern of OPN among different variants of UCA. In the intraluminal cases, OPN immunoreactivity was noted in neoplastic epithelial cells with no peritumoral stromal reaction. This expression pattern was similar to that noted in DC. This finding might explain the favorable behavior of these two subtypes as being non aggressive variants of UCA [28]. In the mural cases, the OPN expression was noted in neoplastic epithelial cells as well as peritumoral stromal tissue. This difference in OPN localization in this particular variant when compared to luminal and intraluminal UCA might explain the difference in the biological behavior of these variants. This explanation is supported by the argument of Wang and Liu [25] who stated that the tumor cell produced OPN could facilitate the tumor cell adhesion and migration in the bone resulting in tumor invasion and spread. So this weak expression of OPN in luminal and intraluminal subtypes, when compared to the mural subtype, might explain why these two subtypes have a favorable prognosis and less invasive behavior than that of the mural subtype.

In MCA, the results of the present study revealed that in both follicular and plexiform ameloblastoma, cytoplasmic OPN immunoreactivity was observed in ameloblast like cells, with cytoplasmic localization in few stellate reticulum like cells. Peritumoral reaction was also noted in the stroma surrounding the tumor. This distribution pattern was found to be consistent with that reported by Wang and Liu [25].This expression pattern of OPN could be explained by the argument of Wang and Liu [25] who stated that OPN protein is probably synthesized and secreted by stellate reticulum like cells, picked up by ameloblast like cells and released into the peritumor nest stromal tissue (transcytosis in ameloblast like cells) in ameloblastoma. They also added the possibility that a small amount of OPN is produced by ameloblast like cells also could not be ruled out [25].

Since OPN can enhance tumor cell migration, invasion and spread, activate osteoclasts, and protect cells from immune mediated cytotoxicity, the elevated expression of OPN in ameloblastoma tumor cells and peritumor nest connective tissue of ameloblastoma, can, at least, partially explain why MCA have the locally invasive behavior and high osteolytic ability [25]. It was reported that OPN expression was associated with recurrence in prostate cancer [22]. Also, odontogenic keratocyst, which is known for its high rate of recurrence, showed strong OPN immunostaining in both its epithelial lining and underlying connective tissue [20]. So, the elevated expression of OPN in ameloblastoma might explain the high recurrence rate of this lesion.

In different lesions included in the present study, OPN expression in inflammatory cells could be explained by the fact induced expression has been detected in T lymphocytes, macrophages, and in remodeling processes such as inflammation, ischemia-reperfusion, bone resorption, and tumor progression [16]. Also, detected OPN expression along the walls of blood vessels might indicate the possible role of OPN in neovascularization. This could be explained by the binding properties of OPN, where the ligation of OPN with integrin α3β5 on vascular endothelial cells induces neovascularization by upregulating endothelial cell migration, survival and lumen formation during angiogenesis. This might explain the OPN immunoreactivity in endothelial cells noted in the present study [16,21]. These angiogenic properties of OPN would increase the rate of tumor growth. The statistical analysis of the present study revealed that the mean area fraction of OPN immunopositivity of UCA and MCA was higher than that of DC. The difference in the mean area fraction of OPN in UCA (mural variant) and MCA when compared to DC might explain the locally invasive behavior of these two variants. This difference was found to be statistically significant. In UCA & MCA, the statistically insignificant difference of OPN expression in these two groups could be due to that most of the UCA studied were of the mural type, as it was reported that the behavior of the mural type is similar to that of the classic intraosseous MCA [28]. Statistical results of the present study revealed a statistically insignificant difference of either CD10 or OPN mean area fraction when comparing recurrent versus non recurrent MCA. This might be due to inconsistent data gathered from a small sample size of the present study. Thus, further studies utilizing more recurrent cases of MCA are highly recommended.

A Pearson's correlation study showed that there was a strong significant positive correlation between the levels of CD10 and OPN expression (R value = 0.622). This correlation was proven to be statistically significant (P value < 0.001). This could be explained by the different biological properties of CD10 regarding neoplastic transformation and tumor progression [9]. OPN enhancement of tumor cell migration, invasion and spread [32], activation of osteoclast [20], and protection of cells from immune mediated cytoxicity [19] might also add to understand this positive association between the two markers. So these two markers could function in a synergistic way to facilitate the neoplastic transformation of DC and the locally invasive behavior of ameloblastoma.

## Conclusions

Based upon the results of the present study, it could be concluded that, in DC, high CD10 and osteopontin expression might predict the neoplastic potentiality of the epithelial lining of this cyst. Also, in UCA & MCA, high CD10 and osteopontin expression might be a useful tool to identify areas with locally invasive behavior and high risk of recurrence.

## Competing interests

The authors declare that they have no competing interests.

## Authors' contributions

SMM participated in the study design, collection of the background references, performing the immunohistochemical technique and photomicrography of the results, writing the discussion of the results and participated in the collection of the background references.

AMA participated in the study design, revised the results and discussion, carried out the statistical analysis.

ESA participated in the study design, revised the image analysis of immunohistochemical results, revised the discussion of the results, participated in the collection of the background references, carried out the sequence alignment, and drafted the manuscript.

All authors read and approved the final manuscript.
